# Anxiety and the impact of the current economic crisis in patients with associated somatic pathology

**DOI:** 10.1192/j.eurpsy.2023.1423

**Published:** 2023-07-19

**Authors:** A.-C. Bredicean, S. Ursoniu, D. Tabugan, L. Palaghian, I. Stoica, S. Tamasan, A. Panfil, A. Neagu, C. Giurgi-Oncu

**Affiliations:** 1 Neurosciences, UMF Timisoara; 2 Psychiatry, SCMUT, Timisoara, Romania; 3 Neurosciences, UMF Timisoara, Timisoara; 4 UMF Timisoara; 5 Laboratory medicine, SCMUT; 6 Psychiatry; 7 Liaison psychiatry, SCJUPBT; 8 Psychologist, SCMUT, Timisoara, Romania

## Abstract

**Introduction:**

Anxiety is a common pathology in people who pass through a crisis situations. Thus, it goes without saying that the current global economic crisis will have secondary effects on mental health.

**Objectives:**

The aim of this study is to identify if there is a correlation between the level of anxiety and the impact of the economic crisis felt patients.

**Methods:**

In this study, 517 patients with known associated somatic pathology were selected. According to gender, 2 groups were formed, that of women (n=308) and that of men (n=209). To identify the level of anxiety, we applied the generalized anxiety disorder assessment scale (TAG7) and to quantify the impact of the global economic crisis felt by the patients, an ordinal scale was used.

**Results:**

190 patients (36.7%) reported that they did not feel anxious at all, while only 24 (4.6%) said that they felt anxiety quite often. Related to the gender, we observed that 36.36% of women and 37.3% of men did not appreciate that they would suffer from anxiety. Those who consider themselves to have high levels of anxiety are represented as follows: 5.8% among the female population and 2.8% among the male population. We researched the correlation between the experienced impact of the global crisis and the state of anxiety and we observed that there is no correlation between the 2 (r=0.19).

**Conclusions:**

The study shows that patients with somatic pathology do not have levels of anxiety correlated with the impact of the global economic crisis felt.

**Disclosure of Interest:**

None Declared

